# Metabolic profiling in *Caenorhabditis elegans* provides an unbiased approach to investigations of dosage dependent lead toxicity

**DOI:** 10.1007/s11306-012-0438-0

**Published:** 2012-06-04

**Authors:** Gita Sudama, John Zhang, Jenefir Isbister, James D. Willett

**Affiliations:** 1 School of Systems Biology, George Mason University, 312A Occoquan Building, PW, MSN: 5B3, 10900 University Boulevard, Manassas, VA 20110 USA; 2 Systems Analytics Inc., Needham, MA 02492 USA

**Keywords:** *Caenorhabditis elegans*, Lead toxicity, Metabolic profiling, Purines

## Abstract

The nematode, *Caenorhabditis elegans* (CE), serves as a model system in which to explore the impact of particularly low-levels of lead [250, 500, 1000 and 2000 parts per million (ppm) (1.4 × 10^−6^ M to 1.1 × 10^−5^ M/nematode)] on specific metabolic pathways and processes. Chromatographic profiles of redox active metabolites are captured through application of high performance liquid chromatography coupled to electrochemical detection (Coularray/HPLC). Principal Component Analysis (PCA: unbiased cluster analysis) and the application of a slicing program, located significant areas of difference occurring within the 2.8–4.58 min section of the chromatograms. It is within this region of the data profiles that known components of the purine pathway reside. Two analytes of unknown structure were detected at 3.5 and 4 min respectively. Alterations in levels of the purine, tryptophan and tyrosine pathway intermediates measured in response to differing concentrations of lead acetate indicate that the effect of lead on these pathways is not linear, yet the ratio of the pathway precursors, tryptophan and tyrosine remains relatively constant. The application of the above combined analytical approaches enhances the value of data generated. Exposure of CE to very low levels of lead produced significant alterations in profiles of electrochemically active compounds.

**Electronic supplementary material:**

The online version of this article (doi:10.1007/s11306-012-0438-0) contains supplementary material, which is available to authorized users.

## Introduction

*Caenorhabditis elegans* (*C. elegans*, *CE*), a free living soil nematode (Hope 1999), serves as an excellent biological system in which to examine some of the attributes of heavy metal toxicity (Boyd et al. 2010; Fowler 2010; Helmcke et al. 2010; Leung et al. 2008; Peterson et al. 2008; Troast and Willett 2008; Roh et al. 2006). Dose dependent alterations in some tryptophan pathway intermediates show linear responses to levels of bioavailable lead (Troast et al. 2007) and correlate with lead toxicity effects observed in this organism (Fowler 2010; Zhang et al. 2010; Wang and Yang 2007; Dengg and van Meel 2004; Boyd and Williams 2003; Anderson et al. 2001).

Lead, a non-degradable heavy metal, is toxic to humans, and particularly hazardous to children, making it an environmental contaminant of special concern. It is a developmental neurotoxicant in the young, with known long-term detrimental effects on learning, memory and behavior (Zhang et al. 2010; Xing et al. 2009). Increased blood lead levels in children in lead contaminated environments are found to correlate with decreased performance on intelligence tests [learning deficits] and long term behavioral abnormalities (Kordas 2010; Lanphear et al. 2005; Needleman et al. 1990). Acute lead intoxication is easily detected, however chronic, low-level intoxication (more common in the general population) represents a greater diagnostic challenge (U.S. EPA 2011; Needleman 2009; Binns et al. 2007; Bellinger 2004; Klaassen 2001).

In vivo and in vitro animal studies indicate that several intrinsic signaling pathways involving calcium, zinc, protein phosphorylation, and guanine nucleotide binding proteins, are altered on exposure to lead (Jomova and Valko 2011; Fowler 2010; Razmiafshari et al. 2001; Bressler et al. 1999; Miller et al. 1990). The effect of lead on protein kinase C (PKC) upstream of extracellular signal-regulated kinase 1 and 2 (ERK1/2) likely involves the receptor/non-receptor tyrosine kinases and the Ras signaling transducer (Wang et al. 2009). The ability of lead to displace other metal ions from protein metal-binding sites dependent upon oxygen and sulfur atom coordination makes this metal of particular concern as a toxicant (Godwin 2001). Neurotoxic studies have shown that heavy metals [including lead (Pb)] directly inhibit sequence-specific DNA binding of zinc finger transcription factors (structural motifs), resulting in adverse cellular effects, using synthetic zinc finger peptides (Razmiafshari et al. 2001). Binding to sulfhydryl groups in proteins involved in organismal responses to oxidative stress is an additional component of lead toxicity (Jomova and Valko 2011). Profiles of the electrochemically active compounds obtained in this study include a significant set of analytes (tyrosine, tryptophan and purine pathways) involved in the maintenance and generation of signals essential to homeostatic controls.

Metabolic profiling provides a means of capturing a molecular signal set representative of the phenotypic state (Kośliński et al. 2011; Hines et al. 2010; Willett et al. 2010; Patkar et al. 2009; Yao et al. 2010; Kaddurah-Daouk et al. 2008; Bundy et al. 2008; Kristal et al. 2007; Fiehn 2002). Metabolomics, the study of changes in metabolites due to lead treatment at a global level, can provide information on which metabolic pathways are most affected by lead exposure in the nematode. Such studies of the impact of lead exposure on the CE model system should reveal which sets of signaling arrays within the organism are most sensitive to this metal’s affects.

The focus of this study was to determine if reproducible, meaningful differences in metabolic profiles are produced as a result of this nematode’s exposure to lead. Alterations were detected in profiles of intermediates of the purine, tyrosine and tryptophan pathways using HPLC with electrochemical detection. Concentrations of tyrosine and tryptophan were monitored as the obligatory precursors to several metabolic pathways examined in CE after exposure to lead. Kynurenine, an intermediate of the tryptophan degradative pathway involved in nicotinamide metabolism, was monitored as an indicator of the effect of lead on one route in energy metabolism (Baranowska-Bosiacka and Hlynczak 2003). Electrochemically active small molecule analyte profiles were generated as chromatograms for untreated and lead acetate treated (0, 0.66 mM, 1.32 mM, 2.64 mM and 5.27 mM lead) 14-day axenic *CE* populations using Coularray^®^ HPLC (HPLC with an electrochemical detector). The chromatographic data were analyzed using CoulArray^®^ software (version 3.0). To increase sensitivity, unbiased and global approaches for data analysis were applied to the chromatographic data: PCA followed by data slicing image analysis using software RAMP (Version 2.0, Systems Analytics Inc., Needham, MA 02492). PCA separated the untreated and lead treated *CE* populations according to dosage (Reich et al. 2008). Analysis of the data image indicated a window of differences that referenced at 2.8–4.58 min in the chromatograms. The analytical techniques used in this research allow detection of subtle changes in *CE* metabolic profiles following exposure to low levels of lead, without referring to neurotoxicant behaviors/end-points.

## Materials and methods

### Nematode strain, growth and lead treatment

*CE* (N2 (Bristol) an axenic strain obtained from Zuckerman (in 1989) was grown and maintained at 21 °C (Precision Low Temperature Incubator 815, #31213, Precision Scientific Inc., Chicago, IL), using a modified version of the axenic culture medium (ACM) and technique (Sayre et al. 1963; Willett et al. 2010). Cultures were grown in 16 ml sterile liquid medium (ACM) and subcultured bi-weekly.

Lead acetate dissolves completely in water whereas lead is insoluble. Hence, lead acetate, a soluble lead compound, was used for *CE* to uptake lead. *CE* displays short term (<12 h) chemotactic responses to acetate (Matsuura et al. 2010). Acetate is used by the nematodes for fatty acids biosynthesis (Rothstein 1972). The media (ACM) the *CE* are grown in contains a surplus of fatty acids, hence the elimination of acetate as a control in this experiment. Different volumes of a sterile stock solution (20 mg/ml or 52.72 mM) of lead acetate (Fisher Scientific # L33-250, Fair Lawn, NJ), were added to *CE* inoculated cultures to achieve lead concentrations of 250, 500, 1000 and 2000 parts per million (ppm) (0.66, 1.32, 2.64 and 5.27 mM). Fifteen *CE* cultures were prepared, with lead concentrations of 0, 250, 500, 1000 and 2000 ppm, in triplicate. Water was added to ensure that the total volume of each flask was 16 ml (of ACM, *CE* and lead acetate). The cultures were maintained at 21 °C, for 14 days. The experiment was repeated under identical conditions. Each culture was given a unique identifier.

### Sample preparation and HPLC analysis

At the end of 14 days, 5 ml *CE* culture from each flask was harvested (Willett et al. 2010), and washed at room temperature to obtain the final pellets containing mixed population *CE* in 300 ml total volume water.

The *CE* pellets were prepared for HPLC analysis (Willett et al. 2010). 600 ml of Mobile Phase A [MPA, an acidic, polar solvent (Willett et al. 2010)] was added to each of the untreated and low level treated (250 ppm) sample pellet. To each of the 500, 1000 and 2000 ppm lead treated sample pellets 300 ml of MPA was added. Volumes of MPA added to the CE pellets varied with pellet size, to reduce the differences in the normalization or scaling constants (soluble protein concentrations) in each sample. Each sample was boiled in a water-bath for 5 min to precipitate the protein, allowed to cool to room temperature and stored at −20 °C, awaiting ultrasonic treatment (Willett et al. 2010) prior to HPLC analysis.

Coulometric array detection coupled with HPLC (CoulArray^®^ HPLC) instrumentation was used to detect and quantify small molecules based on their oxidation–reduction potentials (Matson et al. 1984). This technique has found extensive application in the study of neurotransmitters, and other metabolites derived from biochemical pathways such as those of tyrosine, tryptophan and the purines (Patkar et al. 2009; Yao et al. 2010; Kaddurah-Daouk et al. 2008; Kristal et al. 2007). The CoulArray (CoulArray^®^ Multi-Channel ECD system, ESA Inc., Chelmsford, MA 01824) HPLC system is comprised of two ESA solvent delivery systems (pump Model 582, ESA Inc., Chelmsford, MA 01824), ESA autosampler (Model 540, ESA Inc., Chelmsford, MA 01824), Waters micro Bondapak C_18_ 3.9 × 150 mm Column (Waters Corporation, Milford, MA 01757), ESA 16 channel detectors [4 cells (ESA Inc., Chelmsford, MA 01824)] and is controlled by the CoulArray^®^ for Windows (version 2.0) software. A step gradient solvent flow method with a 70 min run time was created to separate the metabolites from the nematode samples. Data obtained from the samples were in the form of chromatographs, output of the CoulArray^®^ for Windows software.

Each analyte has a characteristic peak shape, time of elution (retention time) and specific oxidation potential profile, dependent upon the instrument and solvent(s) conditions. The chromatograms generated are a representation from electrochemical signals of the redox active compounds separated by HPLC.

Standards consisting of known compounds in defined cocktails (intermediates of purine, tyrosine and tryptophan catabolic pathways), were analyzed at the beginning, during and at the end of each sample batch. The concentration of each compound in the cocktails was not greater than 1 μg/ml. The instrument allows detection of >5 pg/ml of most redox-active substances (Kristal et al. 1998).

*CE* cell lysates from each sample were subjected to HPLC analysis to generate chromatograms (CoulArray^®^ software) and were assayed for total protein content using the Bradford protein assay procedure (BIO-RAD, P123236, Hercules, CA). The protein data were used to normalize the chromatographic data, generated from the CoulArray^®^ HPLC system.

### Flow diagram with data set description

A linear flow diagram of the experiment, data collection and data pre-processing and analysis, was generated in accordance with Goodacre et al. (2007), Fig. 1. It provides an outline of the experimental design, and a summary of the data processing at a glance.

**Fig. 1:**
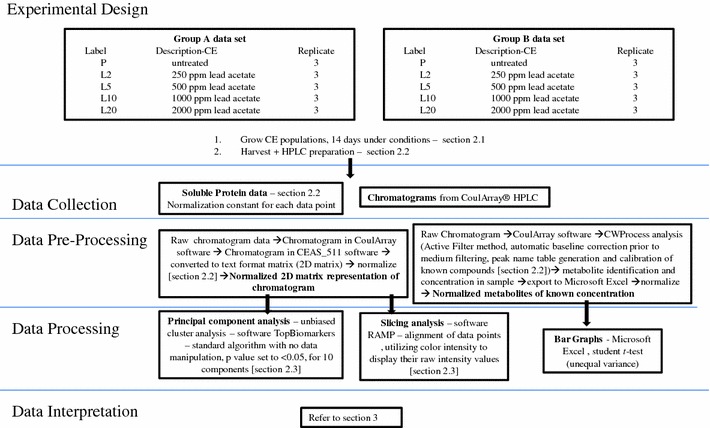
A linear flow diagram identifying the flow of information in the experiment

The effects of lead on CE were studied at levels of 0–2000 ppm lead acetate. Duplicate sets were tagged as A and B respectively for the first and second population groups studied. In each instance, three sets of experiments were conducted, involving: no lead, and the four levels of lead, labeled as 1, 2 or 3 for each of the three subsets from the original population used to populate each component of the study, and the letter code L2, L5, L10 or L20, to indicate levels of lead acetate as; 250, 500, 1000 or 2000 ppm respectively. Controls were designated with the letter P, as 1P or 2P etc. Thus the designation, 1L10A, indicates, the first subset of nematodes, from the first of the seed populations exposed to 1000 ppm lead acetate, while 2L20A, would be the second subset of nematodes, from the first of the seed populations, exposed to 2000 ppm of lead acetate. A sample, 2L5B, would be the second subset of nematodes, from the second seed population (population B) treated at 500 ppm lead acetate. Controls for subsets generated from seed population B, would be designated with the letter PB.

Table S1 shows the data attributes of each group for different dosage levels and Table S2 shows the number of samples used in analysis of each group for different dosage levels, located in the supplementary information (SI). The chromatograms and source text files generated from the chromatograms are located in the supplementary information (Fig. S2 and Tables S3 through S16). Each of these samples was measured using CoulArray^®^ with 16 channels. A control population (sample) from each experiment and one 250 ppm treated sample (from experiment B) were compromised during sample preparation; hence no chromatographic data were obtained from these samples. The data point/chromatogram from each control and 250 ppm treated sample is a representation of the average value of the metabolites present, obtained from a sample of approximately 6,000 nematodes.

As part of the pre-processing process, the chromatograms were converted into number files (2D text format matrix) using a module of the CoulArray^®^ software (Data Module Version 2.0), CEAS_511 software (provided by ESA), a generated Perl script to adjust the file names, and a Python script to normalize the data with the soluble protein data (generated in Sect. 2.2, Willett et al. 2010). The normalized number files matrix has 16 columns (a column for each applied voltage) and 8760 rows (a row represents 0.5 s time line of the data). Following, data analyses were performed on the matrices using software TopBioMarkers (Version 1.5) for PCA analysis and RAMP (Version 2.0) for slicing analysis, both provided by Systems Analytics Inc., Needham, MA 02492. These software were used in The MicroArray Quality Control (MAQC) Consortium for the development and validation of microarray-based predictive models (MAQC Consortium 2010, in the reference).

In addition, pre-processing was performed by the CoulArray^®^ software to obtain concentrations of know metabolites of interest. In the CWProcess module of the software, the chromatographic data undergoes baseline correction and filtering (to reduce the noise), followed by peak identification and concentration. This data is exported to Microsoft Excel where it is normalized against the soluble protein data (generated in Sect. 2.2), for data processing (graphing of data) for interpretation.

### Statistical analysis

Each data point on the PCAs and each chromatogram represent an average of approximately 6000 nematodes exposed to differing concentrations of lead acetate [0, 250, 500, 1000 and 2000 ppm (0, 0.66, 1.32, 2.64 and 5.27 mM lead) or 0–4.3 ppm/nematode or 0–1.1 × 10^−5^ M lead/nematode]. In applying PCA and slicing program to the data, the *p* values were set to <0.05, using unsupervised multivariate linear transformation. The significance (*p*-value <0.05) for the metabolites and metabolite ratios was calculated using Student *t* test (unequal variances), assuming the data is distributed normally. The experiment was performed in triplicate for each lead treatment. Subsequently the entire experiment was replicated. No outliers were removed during data processing.

## Results and discussion

This study explores the impact of low-levels of lead on specific metabolic pathways and processes using CE as a model system. Coularray^®^/HPLC, was used to generate chromatograms representing redox active metabolic compounds, including those found in the purine, tyrosine and tryptophan pathways, followed by PCA to provide unbiased cluster analysis and by application of slicing image analysis to identify major areas of difference in the chromatograms.

### Principal component analysis (PCA)

Each sample chromatogram in Table S1 and Table S2 (see SI) has dimension, 8758 (time points) by 16 (channels, voltage). 2D (dimension) PCA was applied to the chromatographic data in order to gain some unbiased understanding of the clustering of the different samples of varying lead dosage levels, for 10 principal components. The normalized raw data (2D matrices) were used with no other data manipulation, using standard PCA algorithm (see Sect. 2.3).

#### Group A

**Fig. 2:**
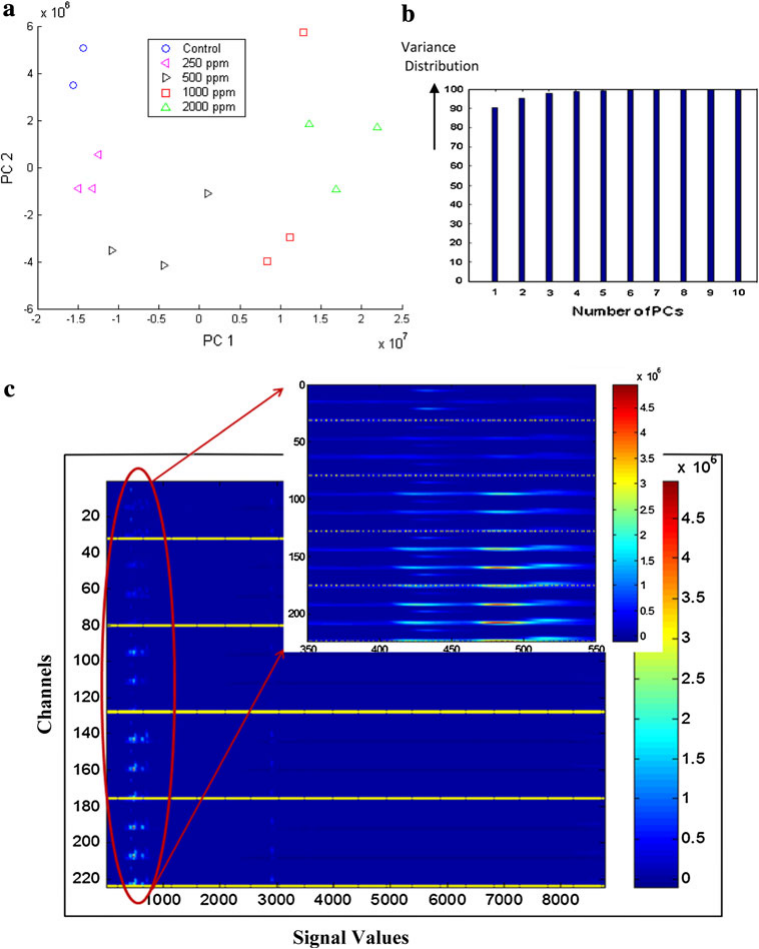
**a**–**c** Representation from data set A. **a** Score plot for the first 2 principal components (PC) for Group A data set. The data are in the form of chromatograms, generated using the technique of HPLC with electrochemical detector, for *CE* populations grown 14 days with five levels of lead acetate (0–2000 ppm). Principal components are vector representations of variable differences in the data set. PC1 captures the largest variable difference and PC2 captures the second largest variable difference. **b** Variance distribution for the first 10 PCs in the data set. **c** Image of Group A data set after application of slicing image analysis. Each horizontal slice is a spectrum for each channel. Each sample has 16 dimensions [y axis (14 samples × 16 channels = 224)], by 8758 [x axis (each unit represents 0.5 s)]. The *yellow lines* show the border among different dose groups, starting with 0 ppm treatment at the *top* and 2000 ppm treatment at the *bottom*. Peak intensity is represented by change in *color* from *blue* (0 intensity) to *red* (maximum intensity, 5 × 10^6^). Differences are seen between 300 and 1000 and, around the 3000 time point. The inset is a magnification of the section with time value 350–550 representing 2.8–4.58 min on the chromatographs. The greatest difference in the data is seen in the 1000 and 2000 ppm data when compared to the untreated nematode populations (Color figure online)

Figure 2a and b show the score plot for the first 2 principal components of Group A dataset (Table S1, in SI) and the variance distribution obtained from the principal component analysis. Score plots are row vectors, obtained from PCA, grouping samples together with similarities in a data set. The following was observed:

- The first two principal components constitute over 95 % total variance of all the principal components, indicating that the use of these two components is enough to capture the main variance information.
- From the score plot, the first principal component score increases with increasing dose level, the score of the second principal component deceases from control to 250 to 500 ppm, then increases from 500 to 1000 to 2000 ppm, reflecting the effect of lead acetate.
- The first principal component counts for about 90 % variance and the dose effect can be mainly observed from the first principal component. In the control and 250 ppm group, little change in the score of the first principal component was observed. The main difference is captured in the second principal component. As the dose level increased, the difference shown on the first PC becomes larger.
- All dose levels are well separated from each other, except for the first sample in the 1000 ppm group. It shows different behavior compared to the other two samples. However, since the first principal component accounts for about 90 % of the variance, the first sample in the 1000 ppm is not very different from the other two samples in the 1000 ppm when looking only at the first principal component.

Figure 2c represents the application of the slicing image analyses to identify major areas of differences in the chromatograms from the five different lead acetate dosage groups in Group A dataset. Peak intensity is represented by change in color from blue (0 intensity) to red (maximum intensity, 5 ´ 10^6^). The horizontal axis represents time of signal capture, and the vertical axis represents channel direction. Every sample has 16 channels: 1–32 represent the control data, 33–80 represent 250 ppm lead acetate treatment data, 81–128 represent 500 ppm lead acetate treatment data, 129–176 represent 1000 ppm lead acetate treatment data, and 177–224 represent 2000 ppm lead acetate treatment. This figure (Fig. 2c insert) also shows the zoom-in image where most of the differences are visual, between 350 and 550 time unit (2.8 and 4.58 min). The image intensity gets brighter with increasing dose level. This data segment (2.8–4.58 min, Fig. 2c insert) may contain potential biomarkers for detecting the level of lead exposure. This section of the chromatogram (from 2.8 to 4.58 min) was found to contain 90–95 % of the metabolic variances following lead exposure, using the slicing image analysis.

#### Group B

Figure S1a (in SI) shows the score plot for first 2 PCs of the group B data set (Table S1 and S2, section SI), Fig S1b (in SI) shows the variance distribution for the first 10 PCs, and Fig. S1c (in SI) presents the image intensity of all the samples in the group B dataset. The following was observed:

- The first 2 principal components account for about 90 % of the variance in the Group B data and capture most of the information in Group B.
- Similar to the analysis for Group A data (Fig. 2), Group B data [Fig. S1 (in SI)] for different dose groups are well separated from each other. With increasing dose level of lead acetate, the score on the first PC tends to increase (from −1.5 × 10^7^ to +1 × 10^7^) and the score on the second PC decreases (from +1.5 × 10^7^ to −1 × 10^7^). There is one overlap in a 1000 ppm and a 2000 ppm dose treatment.

This dataset is used to demonstrate the reproducibility of the experiment and data analysis. Different seed populations were used in each experiment and each experiment was a separate “run” on the CoulArray HPLC. The raw data was used in data analysis with minimum pre-processing. In order to pool the chromatographic data from the entire experiment, to perform PCA, one will need to perform alignment to synchronize the chromatograms (further preprocessing) from different runs.

### Analysis of chromatographic data

**Fig. 3:**
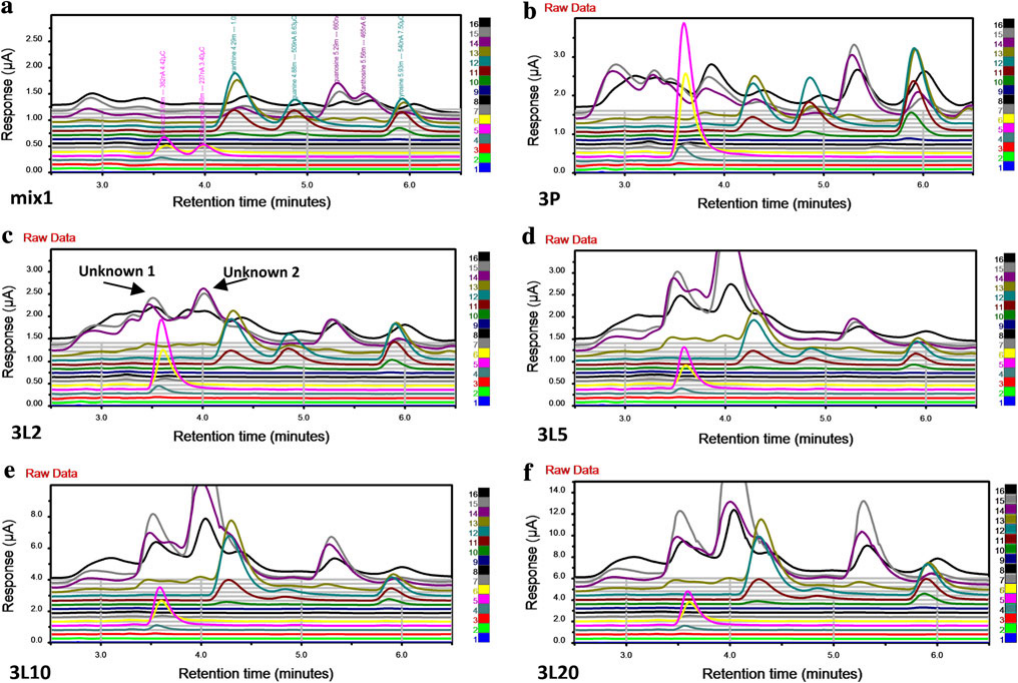
Sections of chromatograms (between retention times 2.5 and 6.5 min) of a cocktail of known metabolites (**a**, mix1), untreated *CE* population cell lysate (**b**, 3P), cell lysate from *CE* populations exposed to 250 ppm (**c**, 3L2), 500 ppm (**d**, 3L5), 1000 ppm (**e**, 3L10) and 2000 ppm (**f**, 3L20) lead acetate, obtained by CoulArray HPLC, using the CoulArray software. **a** Chromatogram of a cocktail of known metabolites (1 mg/ml each). Uric acid eluted at 3.60 min (min) with its primary channel being 5; 8-hydroxy guanine at 3.98 min, channels 5 and 6; xanthine (xan) at 4.28 min, channels 12 and 13; guanine at 4.87 min, channels 11 and 12; guanosine at 5.28 min, channels 14 and 15; xanthosine at 5.56 min, channels 14 and 15; and, tyrosine at 5.93 min, channels 11, 12 and 13. **b** Chromatogram of sample 3PA [untreated 14-day *CE* population (P), the third replicate of set **A**, normalization factor 0.26]. **c** Chromatogram of sample 3L2A [14-day 250 ppm lead acetate treated *CE* population (L2), the third replicate of set **A**, normalization factor 0.41]. **d** Chromatogram of sample 3L5A [14-day 500 ppm lead acetate treated *CE* population (L5), the third replicate of set **A**, normalization factor 0.54]. **e** Chromatogram of sample 3L10A [14-day 1000 ppm lead acetate treated *CE* population (L10), the third replicate of set A, normalization factor 0.63]. **f** Chromatogram of sample 3L20A [14-day 2000 ppm lead acetate treated *CE* population (L20), the third replicate of set A, normalization factor 0.68]. One can see differences when comparing the chromatograms from the untreated to the various levels of lead treated nematode populations

Figure 3a–f are metafiles of the chromatographic outputs from the CoulArray software, of analytes between 2.5 and 6.5 min for a standard [mix1 (Fig. 3a)], untreated [3P (Fig. 3b)] and lead acetate treated *CE* samples [3L2 (Fig. 3c), 3L5 (Fig. 3d), 3L10 (Fig. 3e) and 3L20 (Fig. 3f)]. This region (between 2.5 and 6.5 min) was chosen based on the information obtained from Figs. 2c and S1c **(**in SI). Identification of these chromatographic areas of interest allowed quantitation of these compounds from control and lead acetate treated samples based on elution (or retention) time and primary channel appearance. Two unknown compounds were detected as peaks eluting at 3.5 min (channels 14 & 15) and 4.0 min (channels 14 & 15). These peaks are polar compounds with redox potentials ranging from 780 to 840 mV.

Figure 3 shows sections of 73 min chromatograms runs between 2.5 and 6.5 min. The peaks within the chromatograms of the lead treated populations show significant changes when compared to each other (Fig. 3c–f), even when taking into account the normalization factor (total protein value, Sect. 2.3) listed with the figure. Comparing chromatograms of lead treated samples (Fig. 3c–f) and the untreated sample (Fig. 3b) also show differences. The peaks at 3.5 min (channels 14–15) and 4.0 min (channels 14–15) labeled in Fig. 3c are unknown compounds whose concentrations increased with lead acetate dosage.

**Fig. 4:**
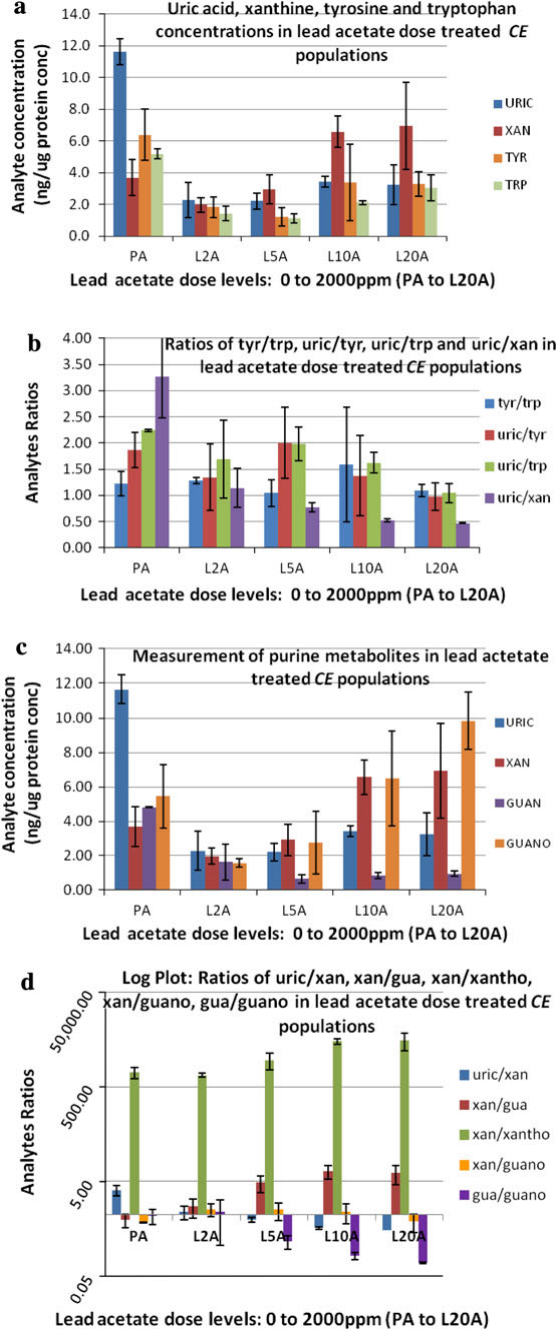
**a**–**d** bar graphs of compounds or ratios of analytes obtained from lead acetate dose treated *CE* populations. The populations were allowed to grow in 0 (PA), 250 (L2A), 500 (L5A), 1000 (L10A) and 2000 (L20A) ppm lead acetate for 14 days. *Abbreviations*: *gua* guanine, *guano* guanosine, *trp* tryptophan, *tyr* tyrosine, *uric* uric acid, *xan* xanthine, and *xantho* xanthosine. **a** Uric, xan, tyr and trp concentrations. **b** Ratios of tyr/trp, uric/tyr, uric/trp and uric/xan. **c** Purine metabolites: uric, xan, guan and guano concentrations. **d** Ratios of uric/xan, xan/gua, xan/xantho, xan/guano and gua/guano, metabolites of the purine pathway

Compounds measured included tyrosine, the amino acid precursor of the catecholamines (dopamine, norepinephrine and epinephrine) and tryptophan, the amino acid precursor of serotonin (a neurotransmitter) and melatonin (a neurohormone) all significant sources of key ligand effectors of components of critical signaling arrays (Blaazer et al. 2008; Kobayashi 2001). Tyrosine eluted closer to the 1000 time unit (retention time 5.93 min) and tryptophan eluted closer to the 3000-time unit (retention time 24.21 min) in Figs. 2c and S1c (located in SI). Also measured were uric acid and xanthine (core components of the purine pathway) which eluted within the window slice extracted from Figs. 2c and S1c (located in SI) data analysis, as well as Fig. 3c, d, f (retention time 2.80–4.58 min). Hence, the concentrations of uric acid (uric), xanthine (xan), tyrosine (tyr) and tryptophan (trp) were monitored following lead treatment and are presented in Fig. 4a.

Uric acid concentrations decreased (from 11.63 ng/mg protein concentration to at least 3.43 ng/mg protein concentration) in all the lead treated populations compared to the control, approximately three-fold [*p* < 0.0014 (Fig. 4a, c)]. Xanthine concentrations fluctuated with lead dosage, decreased in low dose lead treated populations and increased at 1000 and 2000 ppm lead concentrations (twofold (*p* < 0.029)), compared to control CE populations (Fig. 4a, c). Tyrosine and tryptophan concentrations decreased with lead treatment, (about 0.6 fold) when untreated populations were compared with 2000 ppm lead treated populations [*p* < 0.05 (Fig. 4a)]. The ratios of tyrosine/tryptophan (tyr/trp), uric acid/tyrosine (uric/tyr), uric acid/tryptophan (uric/trp) and uric acid/xanthine (uric/xan) are presented in Fig. 4b. Metabolite ratios suggest alterations in flux of metabolites within pathways.

There were no significant changes in the tyrosine/tryptophan (tyr/trp) ratios between untreated and lead treated *CE* populations (Fig. 4b) [data ranging from 0.89 to 1.39 (without the 2.853 outlier in L10A sample which is strongly believe to be chromatographic error), with an average of 1.21 and standard deviation of 0.18] even though both tyrosine and tryptophan concentrations decreased with treatment (Fig. 4a). The uric acid to xanthine (uric/xan) ratio decreased (about sixfold, *p* < 0.0036) with increased lead treatment dosages, up to 1000 ppm treatment (Fig. 4b). This was due more to the decrease of uric acid concentrations than to the fluctuation of xanthine with treatment.

Guanine and guanosine concentrations (metabolites of the purine pathway) also were monitored (Fig. 4c). Guanine eluted at 4.87 min, channels 11 and 12, and guanosine at 5.28 min, channels 14 and 15 (Fig. 3a). Guanosine concentrations fluctuated with treatment. Levels of guanosine in the control samples were greater compared to the 250 ppm treated (L2A) nematode samples by approximately threefold. As lead concentrations increased (greater than 250 ppm), guanosine concentrations increased (from 1.56 ng/mg protein concentration to 9.85 ng/mg protein concentration). Guanine concentrations decreased significantly with treatment (at least twofold, *p* < 0.013). The changes in uric acid, xanthine, guanine and guanosine concentrations in response to lead treatment are presented in Fig. 4c. Uric acid and guanine concentrations decreased significantly with lead treatment when compared to the control, while the concentrations of xanthine and guanosine did not decrease, but rose with the 1000 and 2000 ppm lead treatments.

Data from Fig. 4c is displayed in Fig. 4d as ratios of measured purine metabolites. Figure 4d demonstrates the effects of lead acetate on flux and flow within the purine pathway of *CE*. The ratio of xanthine to guanine (xan/guan) increased by at least threefold (*p* < 0.031) in the 500, 1000 and 2000 ppm 14-day treated *CE* populations, compared to the untreated populations. This is attributed to the dose related increase of xanthine with lead treatment together with the dose related decrease in guanine with lead treatment. A similar pattern is seen with the ratio of xanthine/xanthosine (xan/xantho), which increased by approximately fivefold in the 1000 and 2000 ppm lead acetate, which is contributed by both the dose related increase in xanthine with dose related decrease in xanthosine. Uric acid/xanthine (uric/xan) and guanine/guanosine (gua/guano) ratios resulted in the opposite dose related pattern compared to xan/gua and xan/xantho, with decreases in their ratio from the control to treated populations. Both decreases in ratio values are attributed to the decrease in uric acid and guanine concentrations with altered doses of lead.

**Fig. 5:**
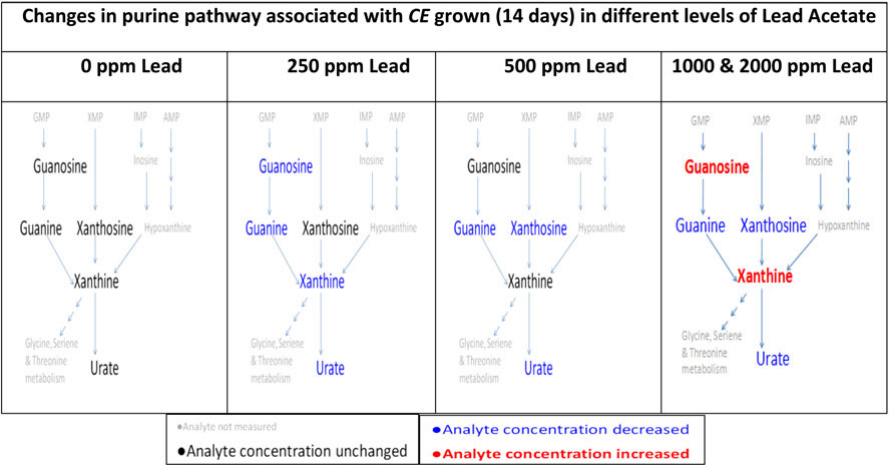
Changes in the purine pathway associated with *CE* grown (14 days) in different levels of lead acetate. The metabolites not measured are grayed-out in smaller font. The measured metabolites from the nematode populations are in *larger black font*. If there is no significant change in the metabolite when compared to the untreated, the color remains unchanged (*black*). If the metabolite concentration is *blue* it denotes a decrease in concentration compared to the untreated nematodes. A *red* metabolite concentration denotes an increase in concentration compared to the untreated nematode (Color figure online)

Decreases in guanine and xanthosine concentrations and the increase in xanthine concentration, with high lead exposure, implies that there are alternate processes affecting purine metabolite levels at high levels of lead exposure. Uric acid concentrations decreased with high lead concentrations (1000 and 2000 ppm) while xanthine concentrations increased, implying that the formation of uric acid from xanthine is affected by lead treatment. In addition, the formation of guanine from guanosine may be reduced by lead treatment, resulting in increasing guanosine and decreasing guanine concentration, with increasing lead dosages. A summary of the changes in purine metabolites after lead treatment of *CE* is presented in Fig. 5.

Figure 5 shows that each treatment of *CE* with varied lead concentrations affected the purine pathway differently. Purines are critical core components of multiple molecular and cellular processes in all living systems affecting functional states of an organism. As single molecular entities, each is known to function as small molecular effectors/ligands, for one or more receptors, ion channels or enzymes that serve to modulate signal transduction processes. They have many important roles and functions in life processes, such as energy transducers, information content and transduction (genes, microRNAs), inter- and intracellular signaling processes, disposal of excess nitrogen etc. For example, nucleotide metabolites, uric acid in particular, have antioxidant properties and can act as neuroprotective or neurotoxic agents (Proctor 2008). In addition, abnormally high uric acid concentrations correlate with gout and cardiovascular diseases while abnormally low uric acid concentrations are associated with neurodegenerative diseases such as Alzheimer’s (Kutzing and Firestein 2008), some of the common recognizable purine metabolic diseases in humans.

All the purine analytes measured were down regulated with treatment at 250 ppm lead acetate while, in the 1000 and 2000 ppm treatment, guanosine and xanthine increased with guanine and uric acid decreasing below the level measured in the 250 ppm treatment. It is possible that uric acid may be acting as a neurotoxicant upon exposure to lead acetate. However, if so, it is showing a non-linear dose response. Micromolar concentrations of lead (Pb) in humans can bind unique RNA motifs (called leadzymes), catalyzing site-specific hydrolysis of the polyribonucleotide chains. These motifs (leadzymes) may be a target for lead (Pb) within the cell, a possible mechanism for destruction of RNA within a cell, leading to lead-mediated toxicity (Barciszewska et al. 2005). In addition, there is a very high degree of specificity with the purine metabolites via purine riboswitches (which discriminates between guanine and adenine by at least 10,000 fold) through simple base pairing to prevent inappropriate gene expression (Gilbert et al. 2009). This may be the reason for the non-linearlity observed in the purine pathway in response to lead toxicity.

Purine nucleotide catabolism proceeds through the formation of xanthine. Xanthine is then converted to uric acid by the enzyme xanathine dehydrogenase/oxidase, prior to excretion. Exposure to lead acetate induces oxidative stress, leading to an increase in xanthine oxidase activity and increased uric acid levels in rats (Soltaninejad et al. 2003), which is seen as well in the 250 ppm lead acetate *CE* populations’ exposure. As the level of lead increases (higher concentrations: 1000 and 2000 ppm) xanthine concentration increases, possibly resulting from gene and protein damage, which may affect the enzymes involve in the purine pathways (Stojilkovic et al. 2010).

Other metabolic response changes observed in *CE* exposed to lead acetate were found in kynurenine (a metabolite in the tryptophan degradative pathway) and in 4-hydroxy benzoic acid (an antioxidant and isomer of salicylic acid). Kynurenine concentration in lead acetate dose treated *CE* decreased by at least half (250 ppm lead acetate treated samples, 2.4 ng/mg protein concentration) (*p* < 0.04) compared of untreated CE populations (0.5 ng/mg protein concentration). This is in accordance with Troast and Willett (2008) findings, a decrease in kynurenine concentration associated with lead acetate treatment. The ratio of tryptophan to kynurenine (trp/kyn) increased by tenfold (*p* < 0.05) in the 1000 and 2000 ppm lead acetate treated populations when compared to the untreated and 250 ppm treated samples. 4-Hydroxy benzoic acid (4HBAC) decreased with lead acetate treatment by at least tenfold (*p* < 0.005) in the 2000 ppm lead acetate treated *CE* when compared to the control. The ratio of tyrosine to 4HBAC (tyr/4hbac) also increased by fourfold (*p* < 0.05) in the 2000 ppm lead treated populations when compared to the control samples. Increases in norepinephrine and epinephrine concentrations with lead acetate treatment correlated with literature findings for lead toxicity in bovine treated cells (Tomsig and Suszkiw 1993).

Breakdown of tyrosine and tryptophan produces second messengers (serotonin and catecholamines). This study showed a decrease in tyrosine, tryptophan and kynurenine concentrations with increasing lead dose. Although tyrosine and tryptophan concentrations decreased in the nematodes with lead treatment, the trp/tyr ratio remained relatively unchanged with treatment suggesting that the mechanisms involved in tyrosine and tryptophan metabolism are affected in the same way by nematode exposure to lead acetate. The decrease in kynurenine and guanine concentrations, both metabolites involved in energy production pathways, mirrored each other. These metabolites are likely participants in lead’s impact on energy metabolism in *CE* and other organisms (Wang et al. 2011; Oleskovicz et al. 2008; Kobayashi 2001).

The availability of tryptophan as a substrate is critical for the synthesis of indole alkyl amines such as serotonin (Fernstrom and Fernstrom 2007). The nuclear tryptophan receptor plays an important role in a sequence of events whereby tryptophan acts to stimulate protein synthesis. Lead acetate was found to inhibit the in vitro binding of l-tryptophan to the hepatic nuclei of mice which have specific receptors for l-tryptophan (Sidransky and Verney 1999) and could limit the availability of tryptophan for the synthesis of serotonin, as seen in our results (Fig. 4a).

## Concluding remarks

Short term exposure to lead at exceptionally low levels (1.1 × 10^−5^ M/nematode) is sufficient to markedly alter measureable metabolic profiles in *C. elegans* as revealed through use of CoulArray^®^/HPLC coupled with PCA and slicing image analysis. The software allows identification of areas of differences resulting from lead exposure when compared to untreated controls. This study of lead toxicity through metabolic profiling, allows identification of subtle changes in the metabolic profiles, that may indicate sensitivity points within the pathways measured and identifies analytes (compounds of interest), which may account for some of the neurotoxic effects of lead. It is evident from these data that lead exposure results in modification of metabolic flux and flow in the pathways whose known metabolites were measured; purine, tryptophan and tyrosine, in comparison to values of these same metabolites observed in unexposed controls. Such modifications within this set of metabolites and the pathways, in which they reside, can result in alterations in the signaling system functions with which these metabolites are involved. Given the import of this particular set of metabolites in neurochemical functions, such modifications are likely participants if not responsible agents in lead’s neurotoxic effects, and its’ known impact on energy metabolism in many organisms (Wang et al. 2011; Oleskovicz et al. 2008; Kobayashi 2001).

Capture of metabolic profiling data may serve as a definitive representation of the phenotypic state of the organism under the specified experimental conditions and is of considerable interest and potential value. The results of such studies are highly dependent upon the data analytic powers of the algorithms utilized in the processing of the data obtained. Application of the above combined analytical approaches enhances the value of data generated. The metabolite profiling output was sufficient to reproducibly track dose responses of the organism to low levels of bioavailable lead [0.5–4.3 ppm/nematode (1.4 × 10^−6^ to 1.1 × 10^−5^ M/nematode)].

## Electronic supplementary material

Below is the link to the electronic supplementary material.

Supplementary material 1 (DOC 156 kb)

Supplementary material 2 (DOC 15000 kb)

Supplementary material 3 (DOC 46 kb)

Supplementary material 4 (DOC 28 kb)

Supplementary material 5 (TXT 868 kb)

Supplementary material 6 (TXT 867 kb)

Supplementary material 7 (TXT 869 kb)

Supplementary material 8 (TXT 870 kb)

Supplementary material 9 (TXT 870 kb)

Supplementary material 10 (TXT 868 kb)

Supplementary material 11 (TXT 868 kb)

Supplementary material 12 (TXT 870 kb)

Supplementary material 13 (TXT 866 kb)

Supplementary material 14 (TXT 866 kb)

Supplementary material 15 (TXT 867 kb)

Supplementary material 16 (TXT 866 kb)

Supplementary material 17 (TXT 865 kb)

Supplementary material 18 (TXT 863 kb)

## Abbreviations

ACM: Axenic culture medium
CE: *Caenorhabditis elegans*
Conc: Concentration
dl: Deciliter
guan: Guanine
guanos: Guanosine
HPLC: High performance liquid chromatography
4-HBAC: 4-Hydroxy benzoic acid
kyn: Kynurenine
M: Molar
mM: Millimolar
mg: Milligram
ml: Milliliter
μl: Microliter
PCA: Principal component analysis
pg: Picogram
ppm: Parts per million
*p* value: Significance
SI: Supplementary information
*t* test: Variance
trp: Tryptophan
tyr: Tyrosine
xan: Xanthine
xantho: Xanthosine
