# Supplementary material for: Metabolic profiling in Caenorhabditis elegans provides an unbiased approach to investigations of dosage dependent lead toxicity
Source: Metabolomics. 2012 Jun 4;9(1):189–201. doi: 10.1007/s11306-012-0438-0 (PMC3548106; doi:10.1007/s11306-012-0438-0)
Supplement: Supplementary file 1 — Supplementary material 1 (DOC 156 kb) [file 11306_2012_438_MOESM1_ESM.doc]

| **S1a**  **1a** | Variance  distribution  **S1b**  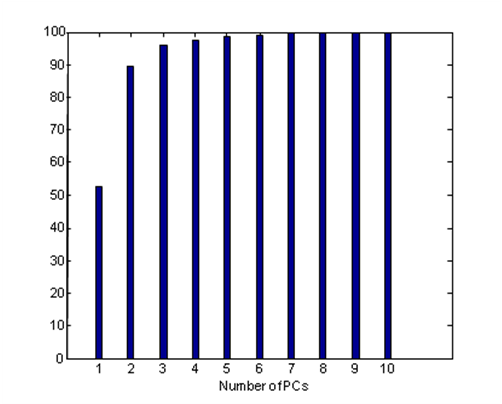 |
| --- | --- |
| **Channels**  **S1c**  **Signal Values** | |

Fig**. S1a, S1b, S1c** (in SI) - representation from data set B. Fig. **S1a** is a score plot for the first 2 PCs (principal components, [vector representation of variable differences in the data set]). The data are in the form of chromatograms, generated using the technique of HPLC with electrochemical detector, for CE populations grown 14 days with five levels of lead acetate dosage (0-2000 ppm). PC1 captures the largest variable difference and PC2 captures the second largest variable difference. Fig **S1b** illustrates the variance distribution for the first 10 PCs in this data set. PC 2 represents the sum of PC 1 and 2, while PC 3 represents the sum of PC 1, 2, and 3. Fig. **S1c** presents an image of Group B data after application of slicing image analysis. Each horizontal slice is a spectrum for each channel. Each sample has 16 dimensions (y axis [14 samples x 16 channels = 224]), by 8758 (x axis [each unit represents 0.5 second]). The yellow lines show the border among different dose groups, starting with 0 ppm treatment at the top and 2000 ppm treatment at the bottom. Peak intensity is represented by change in color from blue (0 intensity) to red (maximum intensity, 5x10^6^). Differences are seen between 300 and 1000 and, around the 3000 time point.
