# Supplementary material for: Metabolic profiling in Caenorhabditis elegans provides an unbiased approach to investigations of dosage dependent lead toxicity
Source: Metabolomics. 2012 Jun 4;9(1):189–201. doi: 10.1007/s11306-012-0438-0 (PMC3548106; doi:10.1007/s11306-012-0438-0)

Fig. S2 – Raw chromatogram files displayed using CoulArray Software. Each display is in .jpeg format.

1PA – Raw chromatogram file displayed using CoulArray Software


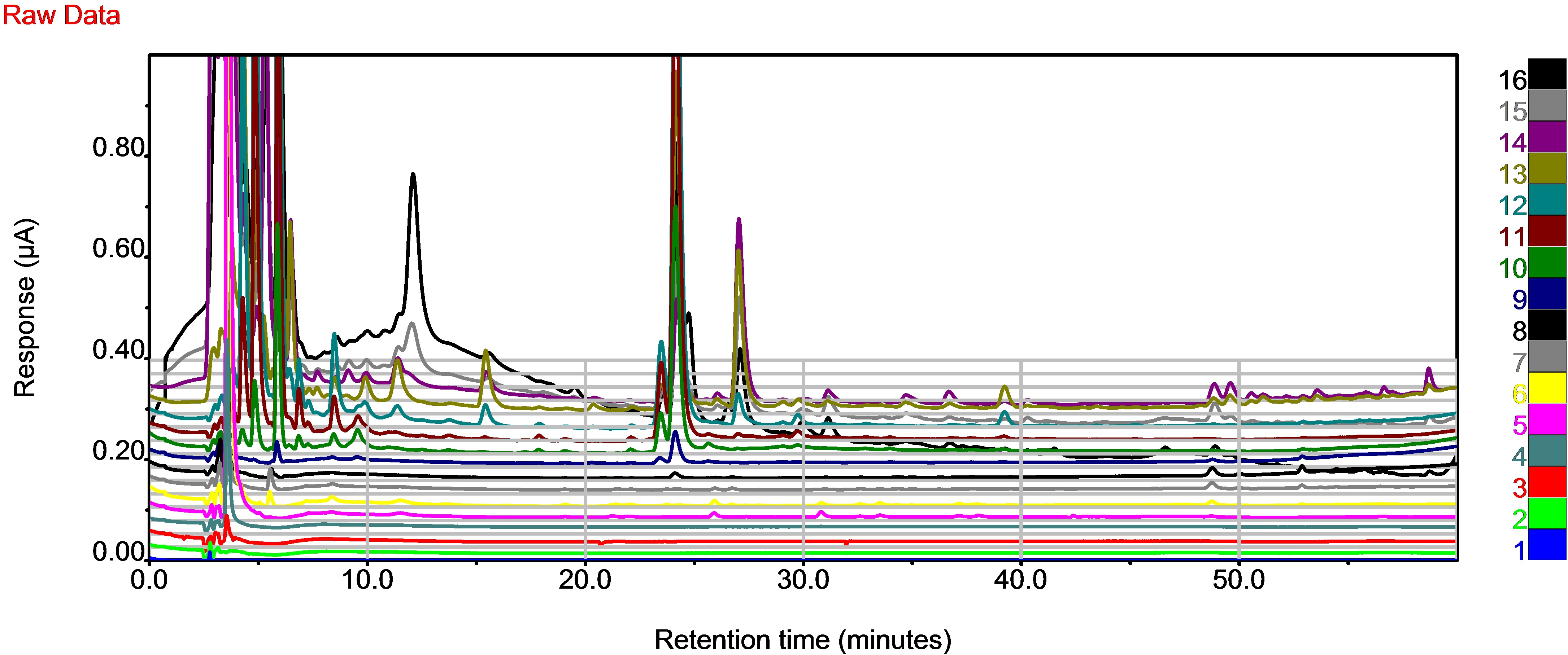


3PA – Raw chromatogram file displayed using CoulArray Software


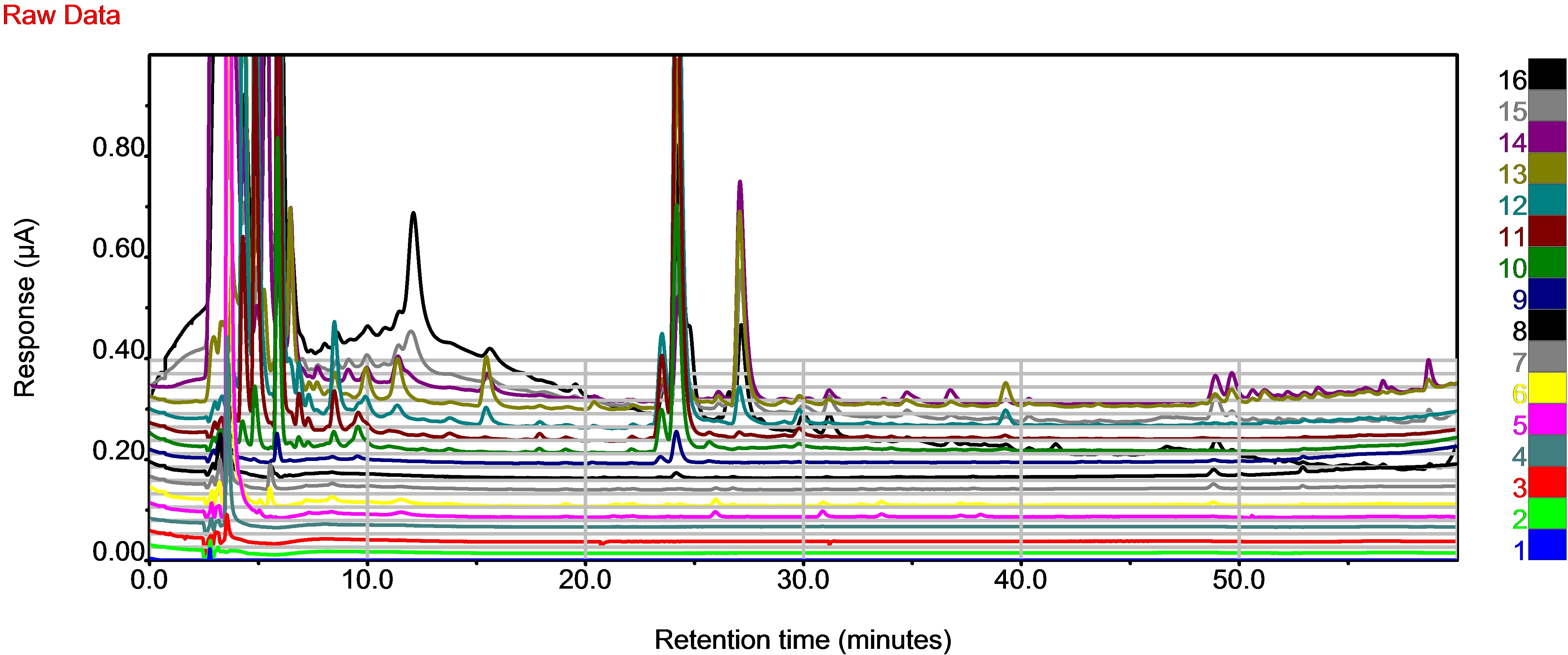


1L2A – Raw chromatogram file displayed using CoulArray Software


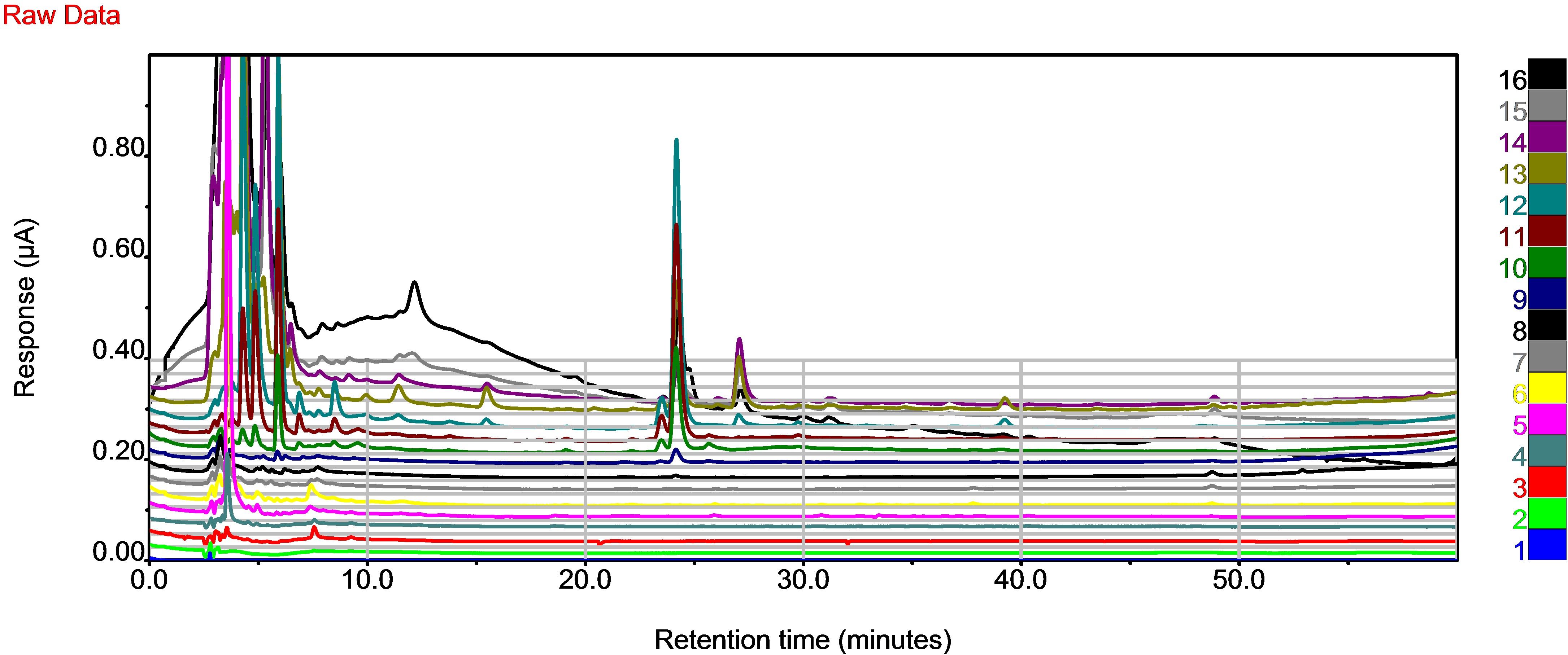


2L2A – Raw chromatogram file displayed using CoulArray Software


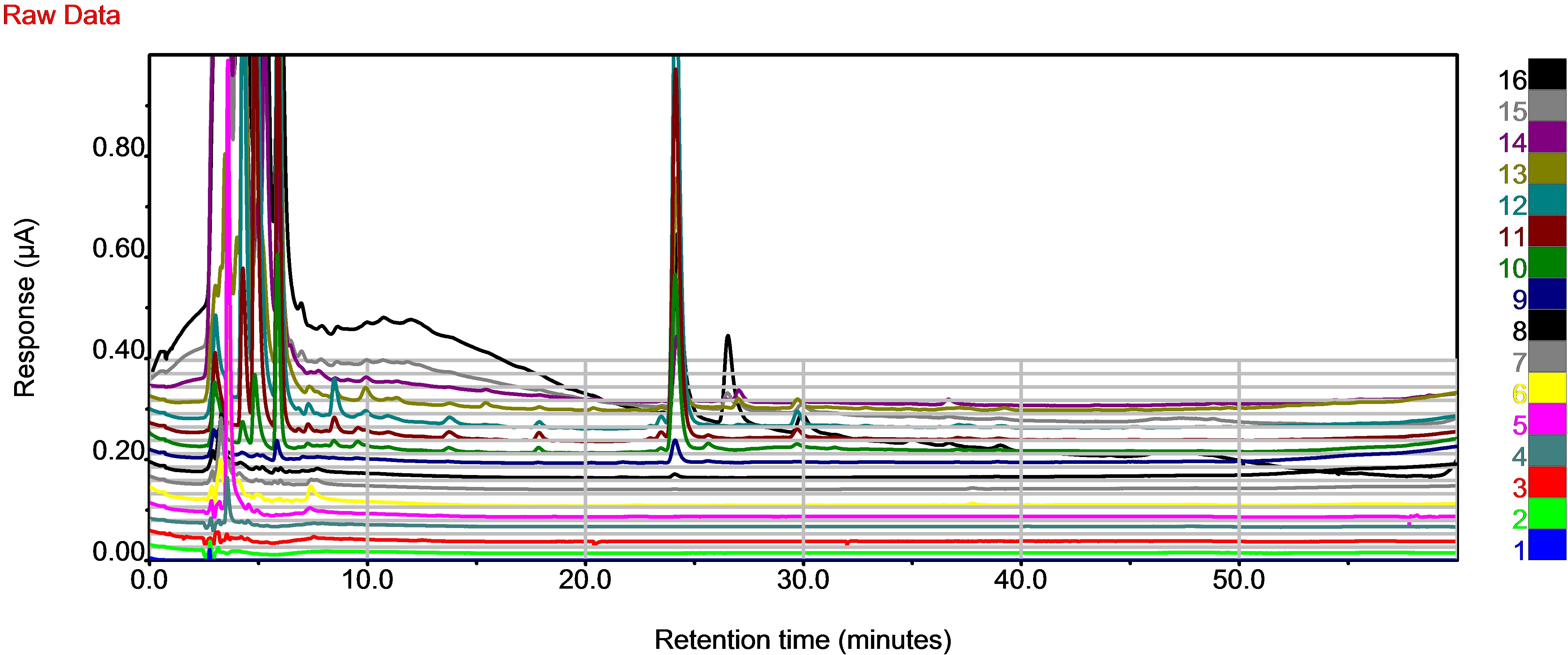


3L2A – Raw chromatogram file displayed using CoulArray Software


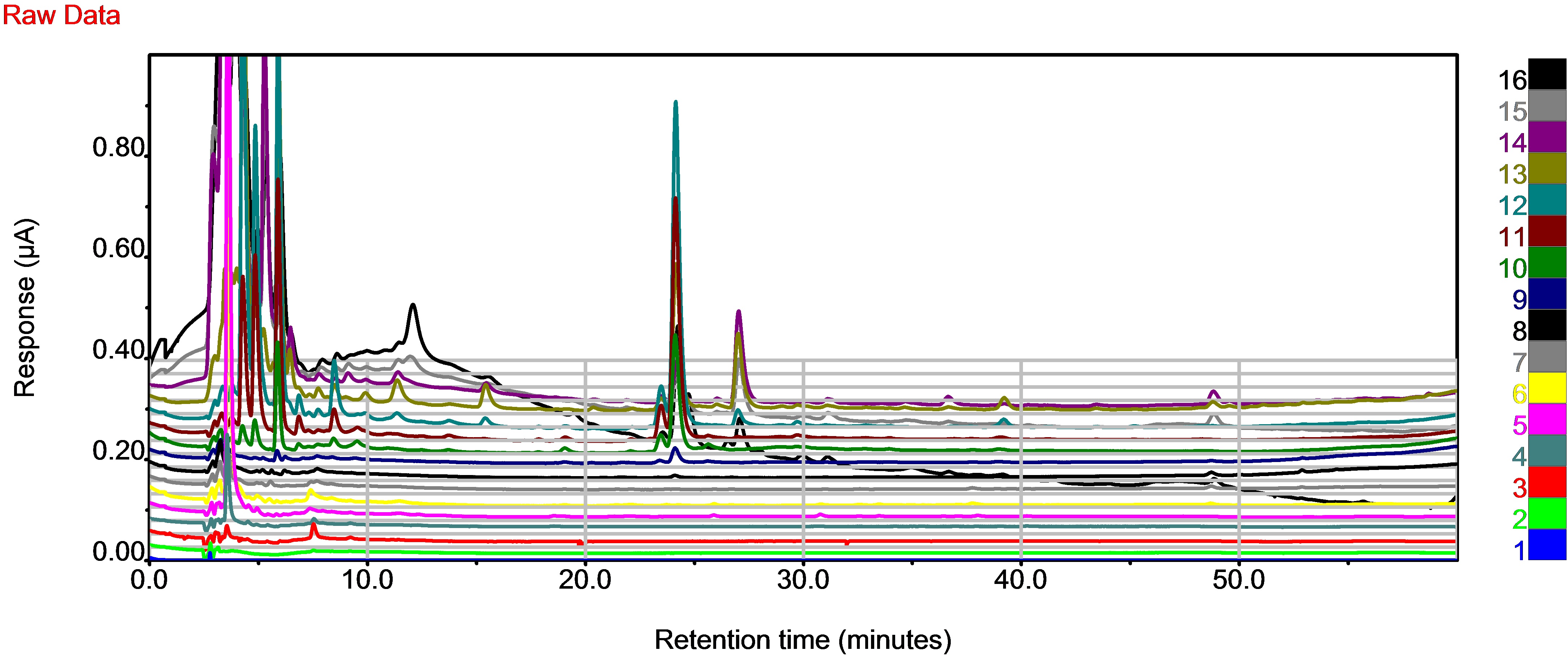


1L5A – Raw chromatogram file displayed using CoulArray Software


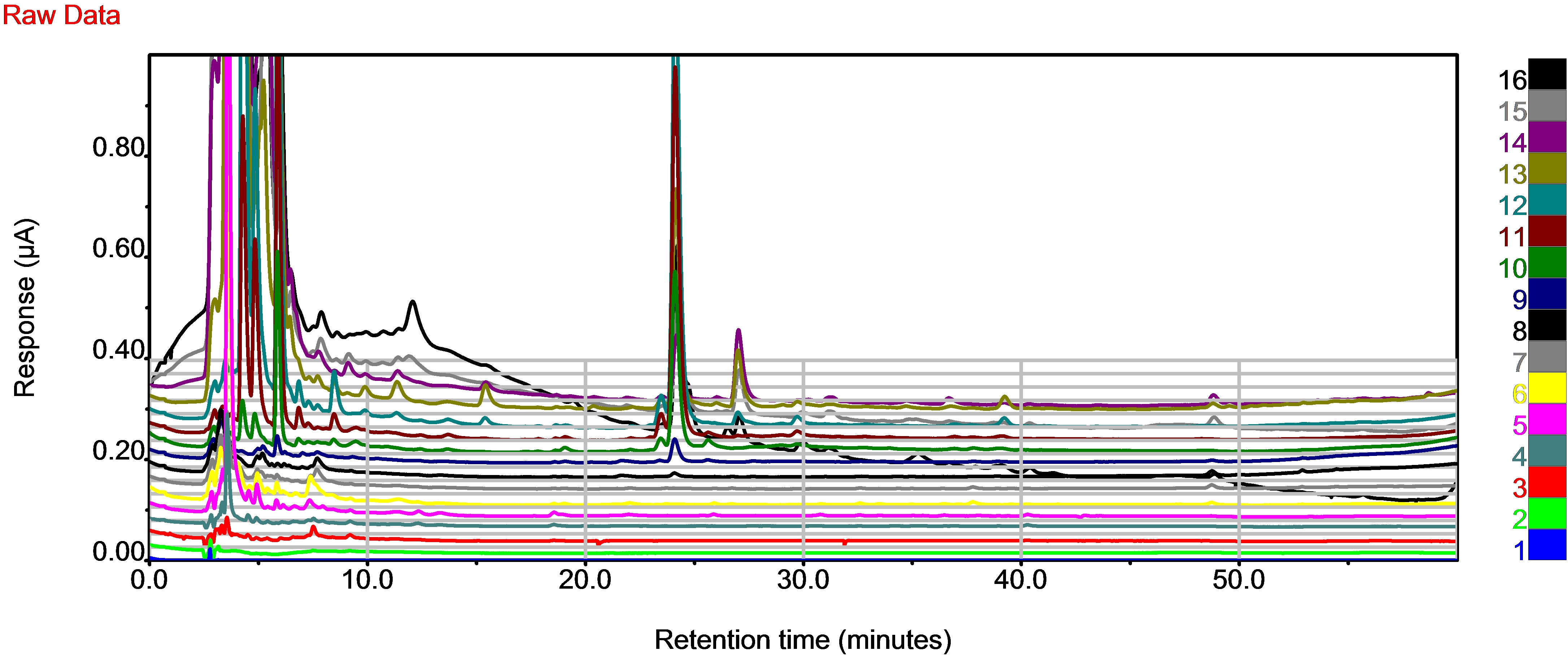


2L5A – Raw chromatogram file displayed using CoulArray Software


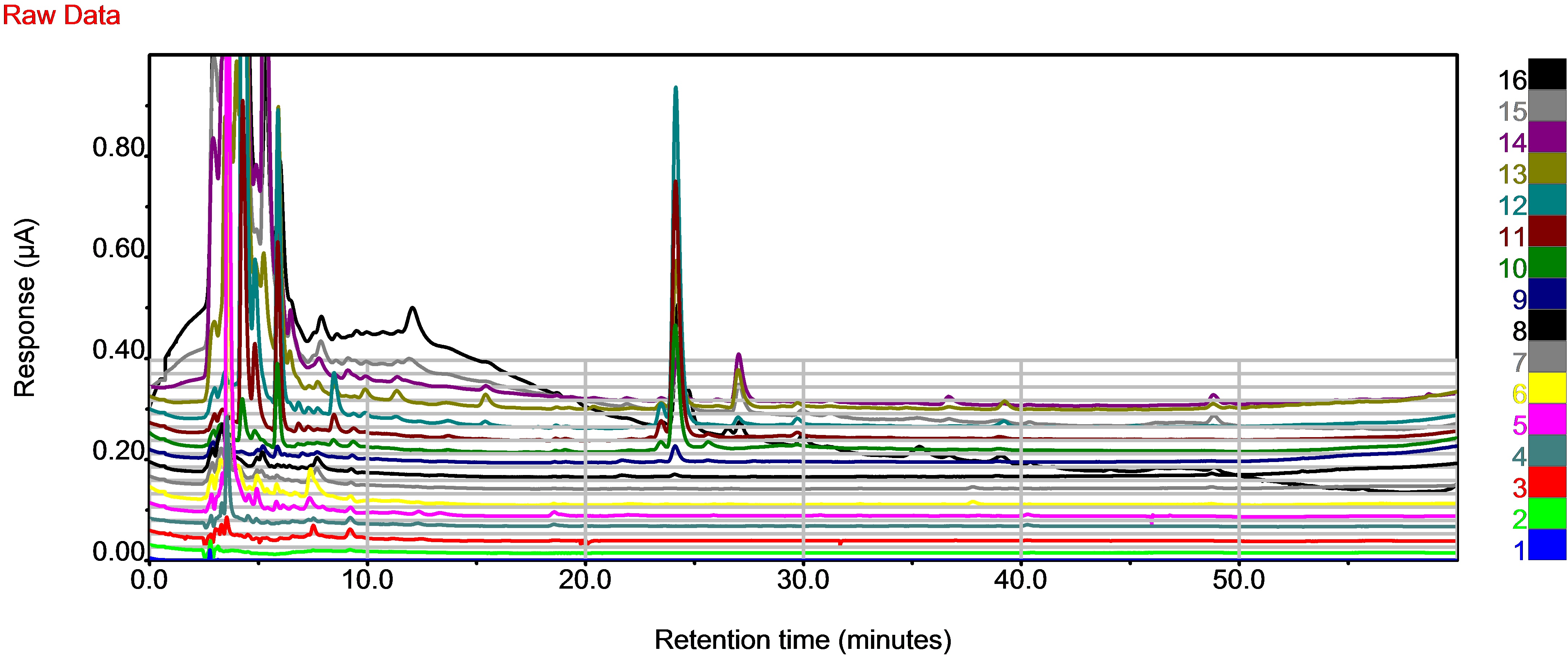


3L5A – Raw chromatogram file displayed using CoulArray Software


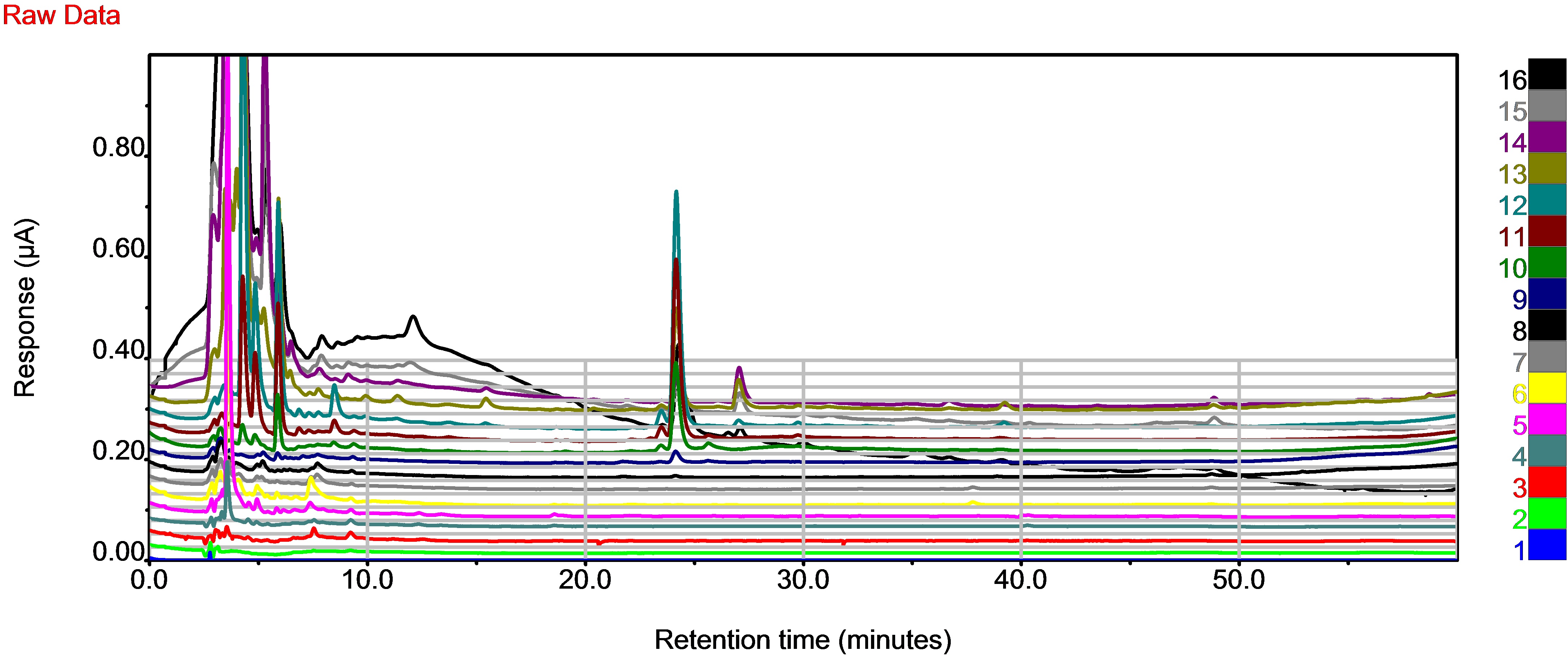


1L10A – Raw chromatogram file displayed using CoulArray Software


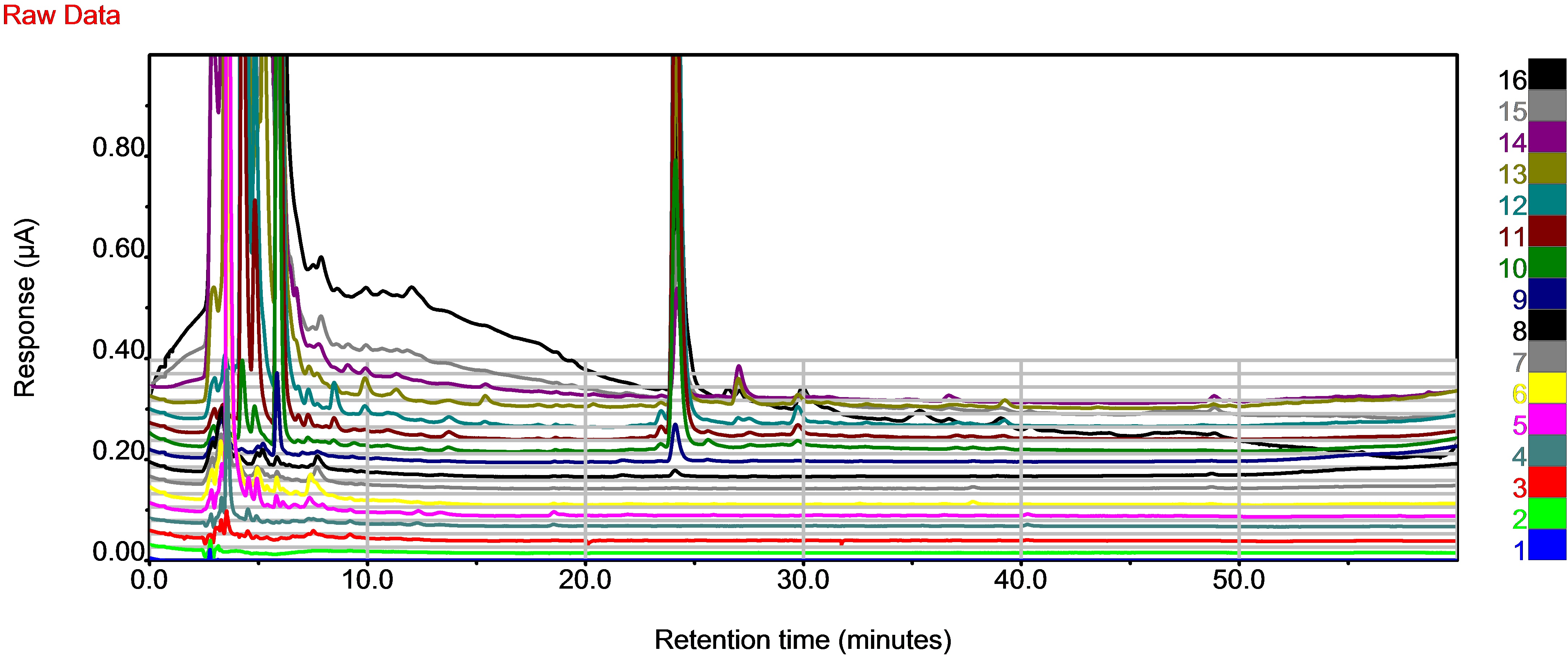


2L10A – Raw chromatogram file displayed using CoulArray Software


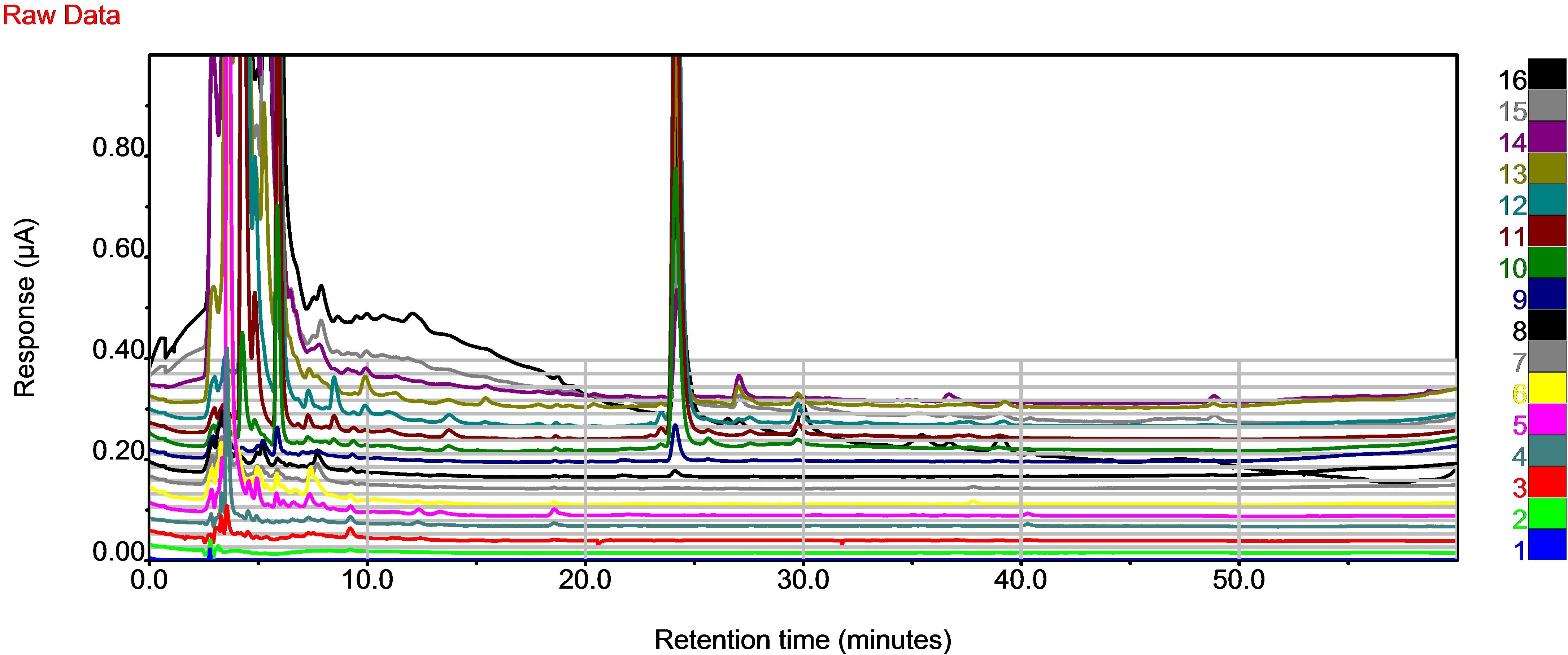


3L10A – Raw chromatogram file displayed using CoulArray Software


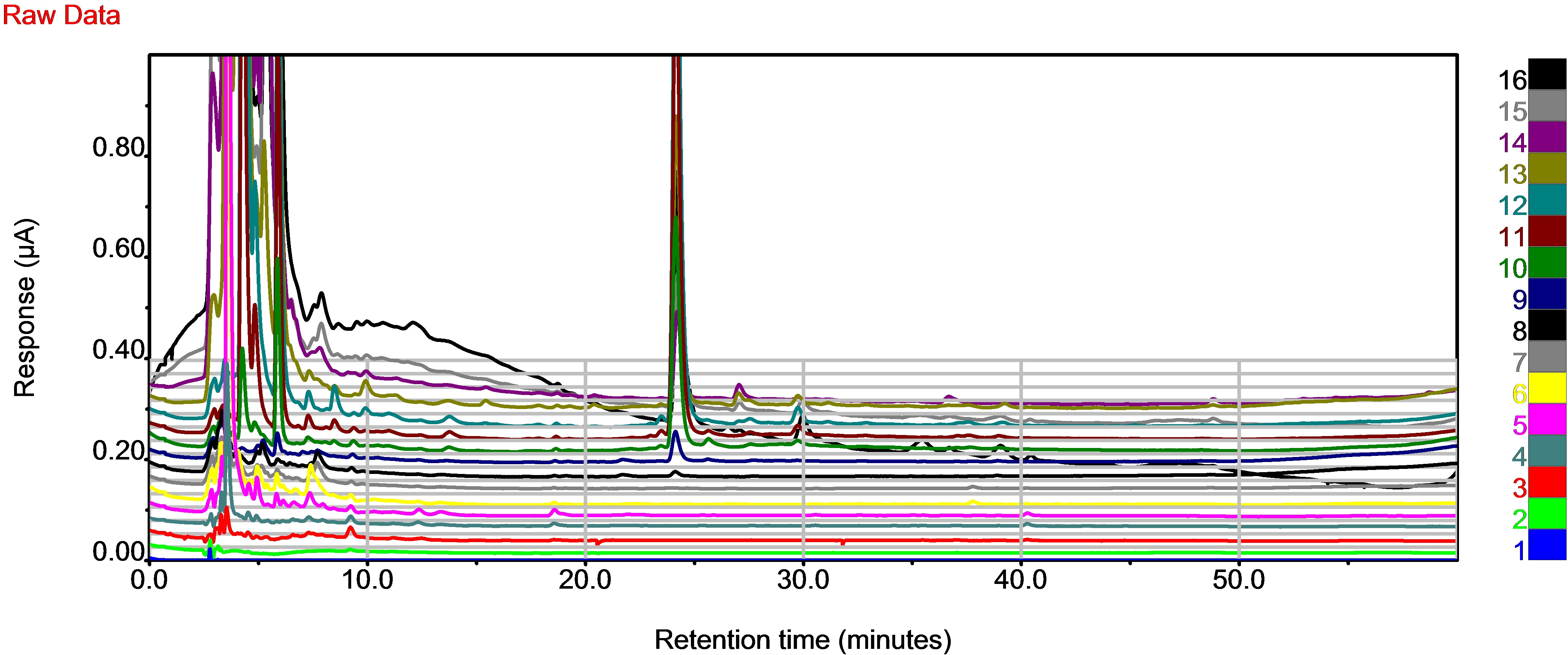


1L20A – Raw chromatogram file displayed using CoulArray Software


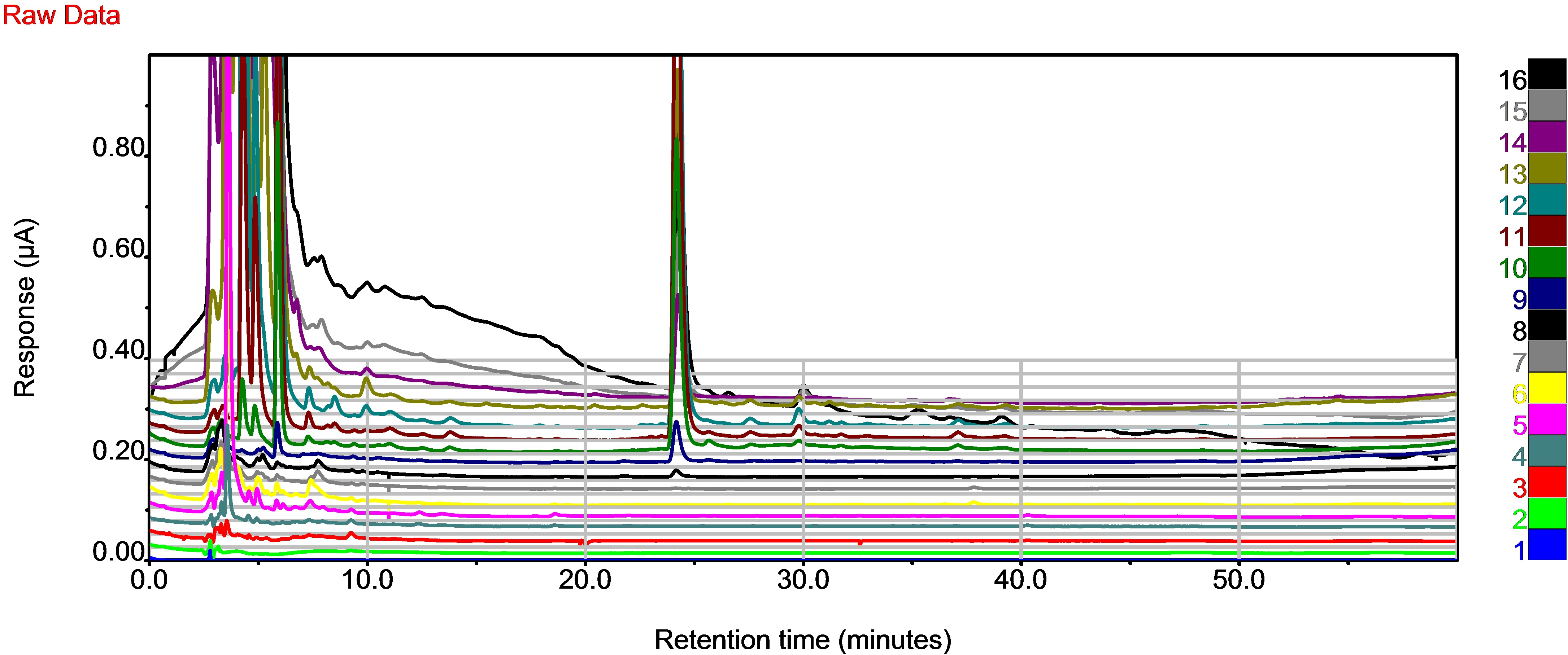


2L20A – Raw chromatogram file displayed using CoulArray Software


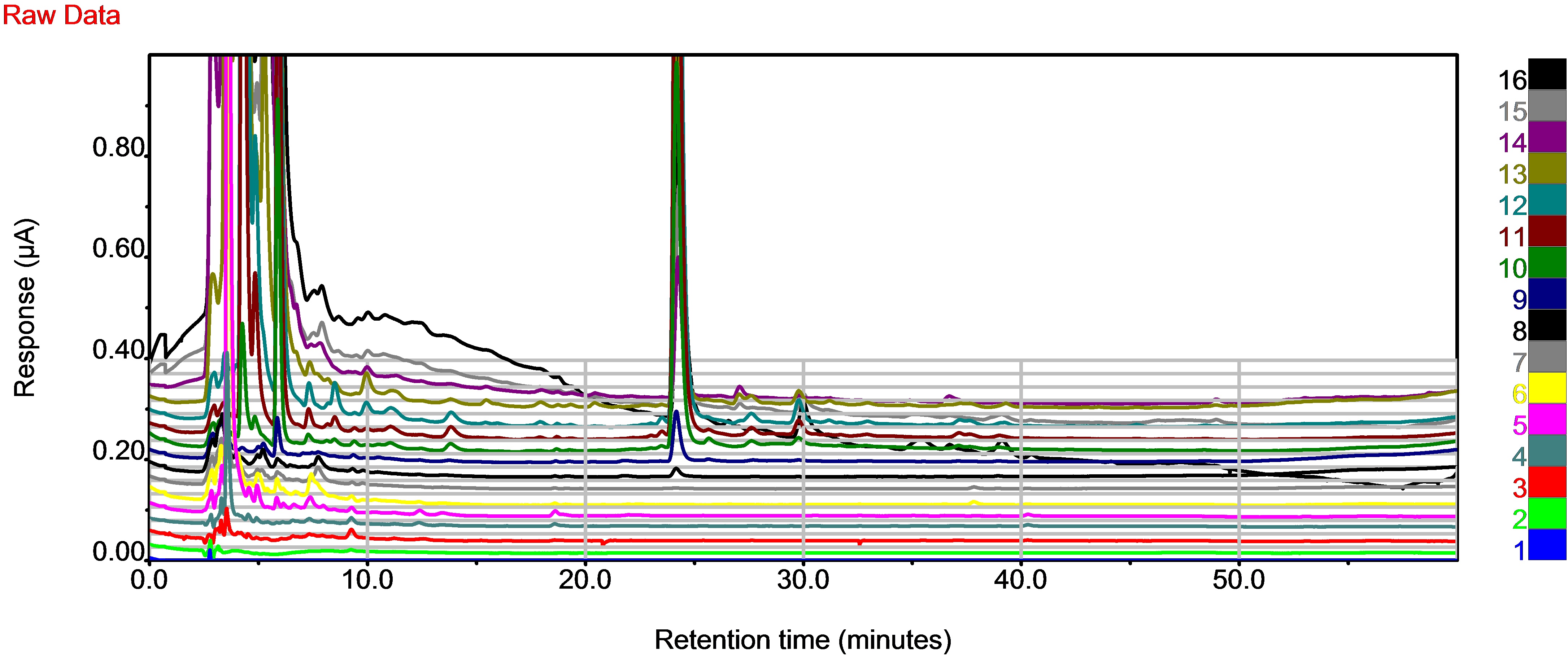


3L20A – Raw chromatogram file displayed using CoulArray Software


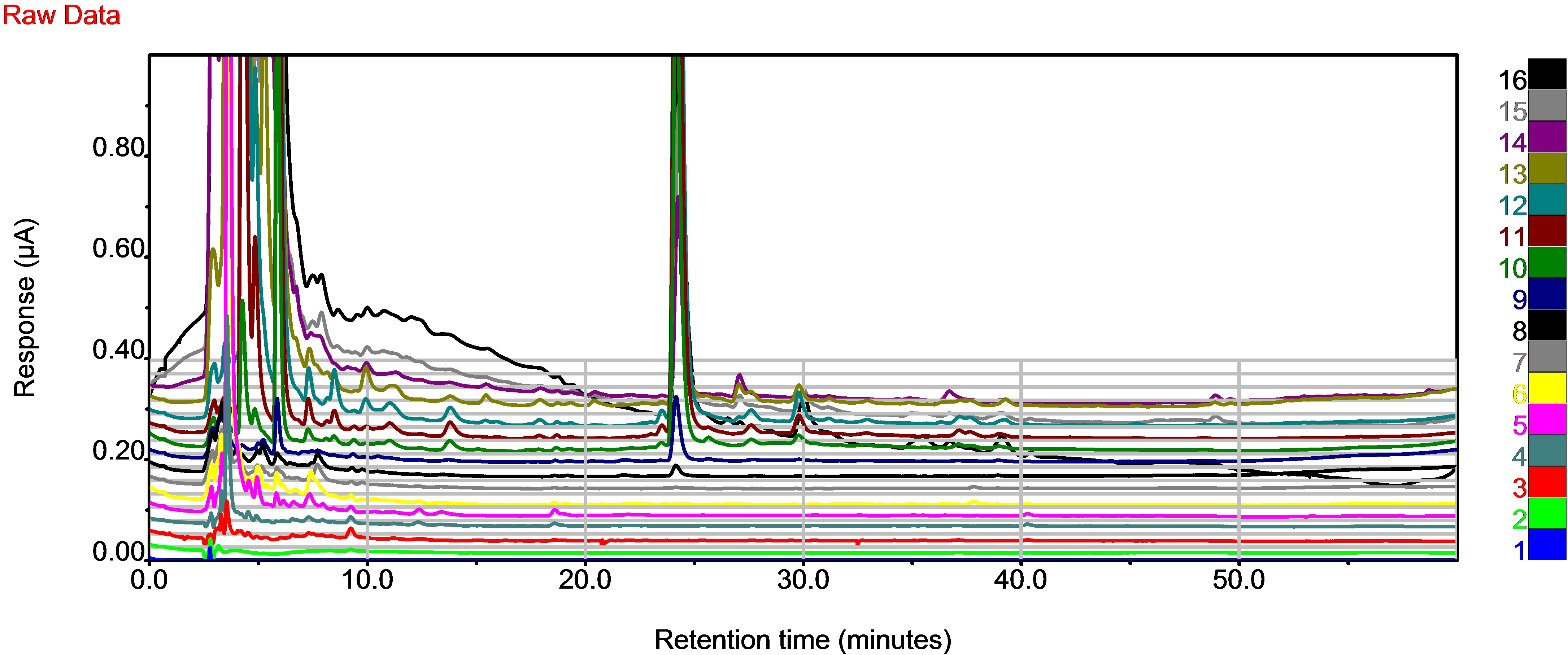

Supplement: Supplementary file 2 — Supplementary material 2 (DOC 15000 kb) [file 11306_2012_438_MOESM2_ESM.doc]
