# Supplementary material for: Metabolic profiling in Caenorhabditis elegans provides an unbiased approach to investigations of dosage dependent lead toxicity
Source: Metabolomics. 2012 Jun 4;9(1):189–201. doi: 10.1007/s11306-012-0438-0 (PMC3548106; doi:10.1007/s11306-012-0438-0)
Supplement: Supplementary file 3 — Supplementary material 3 (DOC 46 kb) [file 11306_2012_438_MOESM3_ESM.doc]

Table S1: Data attributes for Nematodes with different dosage levels of lead acetate. The sample descriptions are the names of the samples of the various treatments.

| Sample Description | Toxic Material | **Dose(ppm)** | Population | **Category** |
| --- | --- | --- | --- | --- |
| 1L20A | Lead acetate | 2000 | 1 | **A** |
| 2L20A | Lead acetate | 2000 | 2 | **A** |
| 3L20A | Lead acetate | 2000 | 3 | **A** |
| 1L10A | Lead acetate | 1000 | 1 | **A** |
| 2L10A | Lead acetate | 1000 | 2 | **A** |
| 3L10A | Lead acetate | 1000 | 3 | **A** |
| 1L5A | Lead acetate | 500 | 1 | **A** |
| 2L5A | Lead acetate | 500 | 2 | **A** |
| 3L5A | Lead acetate | 500 | 3 | **A** |
| 1L2A | Lead acetate | 250 | 1 | **A** |
| 2L2A | Lead acetate | 250 | 2 | **A** |
| 3L2A | Lead acetate | 250 | 3 | **A** |
| 1PA | Lead acetate | 0 | 1 | **control, A** |
| 2PA | Lead acetate | 0 | 2 | **control, A (spilled)** |
| 3PA | Lead acetate | 0 | 3 | **control, A** |
| 1L20B | Lead acetate | 2000 | 1 | **B** |
| 2L20B | Lead acetate | 2000 | 2 | **B** |
| 3L20B | Lead acetate | 2000 | 3 | **B** |
| 1L10B | Lead acetate | 1000 | 1 | **B** |
| 2L10B | Lead acetate | 1000 | 2 | **B** |
| 3L10B | Lead acetate | 1000 | 3 | **B** |
| 1L5B | Lead acetate | 500 | 1 | **B** |
| 2L5B | Lead acetate | 500 | 2 | **B** |
| 3L5B | Lead acetate | 500 | 3 | **B** |
| 1L2B | Lead acetate | 250 | 1 | **B** |
| 2L2B | Lead acetate | 250 | 2 | **B (compromised)** |
| 3L2B | Lead acetate | 250 | 3 | **B** |
| 1PB | Lead acetate | 0 | 1 | **control, B** |
| 2PB | Lead acetate | 0 | 2 | **control, B (spilled)** |
| 3PB | Lead acetate | 0 | 3 | **control, B** |
