# Supplementary material for: Metabolic profiling in Caenorhabditis elegans provides an unbiased approach to investigations of dosage dependent lead toxicity
Source: Metabolomics. 2012 Jun 4;9(1):189–201. doi: 10.1007/s11306-012-0438-0 (PMC3548106; doi:10.1007/s11306-012-0438-0)
Supplement: Supplementary file 4 — Supplementary material 4 (DOC 28 kb) [file 11306_2012_438_MOESM4_ESM.doc]

Table S2. The number of samples analyzed, of each group, in different dosage levels.

| Dosage Level (ppm) | A | B |
| --- | --- | --- |
| Control | 2 | 2 |
| 250 | 3 | 2 |
| 500 | 3 | 3 |
| 1000 | 3 | 3 |
| 2000 | 3 | 3 |
